# A training and education program for genome medical research coordinators in the genome cohort study of the Tohoku Medical Megabank Organization

**DOI:** 10.1186/s12909-019-1725-5

**Published:** 2019-08-02

**Authors:** Mika Sakurai-Yageta, Hiroshi Kawame, Shinichi Kuriyama, Atsushi Hozawa, Naoki Nakaya, Fuji Nagami, Naoko Minegishi, Soichi Ogishima, Takako Takai-Igarashi, Inaho Danjoh, Taku Obara, Mami Ishikuro, Tomoko Kobayashi, Yayoi Aizawa, Rino Ishihara, Masayuki Yamamoto, Yoichi Suzuki

**Affiliations:** 1 Tohoku Medical Megabank Organization, Tohoku University, 2-1 Seiryo-machi, Aoba-ku, Sendai, Miyagi 980-8573 Japan; 2 Graduate School of Medicine, Tohoku University, 2-1 Seiryo-machi, Aoba-ku, Sendai, Miyagi 980-8575 Japan; 3 International Research Institute of Disaster Science, 468-1 Aramaki Aza-Aoba, Aoba-ku, Sendai, Miyagi 980-8572 Japan; 4 Ageo Central General Hospital, 1-10-10 Kashiwaza, Ageo, Saitama, 362-8588 Japan

**Keywords:** Genome cohort study, Biobank, Informed consent, Genome medical research coordinator, Education

## Abstract

**Background:**

Genome cohort studies are used to analyze interactions between genetic and environmental factors, providing valuable information for personalized healthcare. Large-scale and long-term cohort studies require a number of specially trained personnel, of whom those involved in obtaining informed consent play a vital role, especially during the initial phase of such studies. The Japanese Society of Human Genetics (JSHG) previously established a certification system for genome medical research coordinators (GMRCs) responsible for obtaining written consent via face-to-face explanation. Meanwhile, in the Tohoku Medical Megabank Organization (ToMMo), GMRCs are expected to play important roles not only in obtaining informed consent and conducting various assessments, but also in communicating with participants throughout the long-term follow-up. Based on the JSHG program, we therefore developed a specific education and training program for ToMMo GMRCs consisting of 17 lectures, one practical training session on the informed consent procedure, and written and interview examinations. Re-education workshops aimed at self-improvement are also carried out following certification. In this study, we evaluated the education and training program in terms of overall understanding, usefulness, and satisfaction using an anonymous questionnaire.

**Methods:**

An anonymous questionnaire addressing each aspect of the education and training program (understanding, usefulness, and satisfaction) was distributed among 152 qualified ToMMo GMRCs. Responses were received from 94 participants (61.8%).

**Results:**

There was a significant association between the level of overall understanding of lectures and medical qualification (nurse or clinical laboratory technologist), but not with age or educational background. The level of understanding and overall usefulness were lower in sessions related to genetics and epidemiology than those dealing with ToMMo practices. In the re-education workshops, GMRCs showed a preference for and hoped to learn more about both background knowledge and research progress in the ToMMo.

**Conclusions:**

The results of our questionnaire suggest that not all ToMMo GMRCs are able to understand everything during the initial education and training program, especially in terms of genomic medicine. Continuous re-education is therefore vital in improving knowledge, skills and motivation, and preparing GMRCs for a specialist role in community-based personalized healthcare.

**Electronic supplementary material:**

The online version of this article (10.1186/s12909-019-1725-5) contains supplementary material, which is available to authorized users.

## Background

The Tohoku Medical Megabank Project (TMM) was launched to support creative reconstruction following the Great East Japan Earthquake in March 2011, providing medical support and carrying out a genome cohort study [1–3]. Large-scale prospective cohort studies and/or biobank projects are necessary for analysis of gene-environment interactions and the subsequent development of genomic medicine and personalized healthcare [4]. The TMM project, which aims to examine the fundamental research infrastructure of Japan, is being conducted by two organizations, Tohoku University Tohoku Medical Megabank Organization (ToMMo) and Iwate Medical University’ Iwate Tohoku Medical Megabank Organization (IMM). A total of 150,000 residents were recruited in fiscal year (FY) 2013–2016.

As part of the TMM project, the ToMMo constructed two cohorts consisting of more than 120,000 participants: the TMM Community-Based Cohort (TMM CommCohort) study, which includes 50,000 adults living in Miyagi prefecture, and the TMM Birth and Three-Generation Cohort (TMM BirThree Cohort) study, which consists of 70,000 family members of pregnant women. Meanwhile, the IMM constructed a TMM CommCohort consisting of more than 30,000 participants. In the ToMMo studies, assessments were conducted as follows: (1) onsite during annual community health examinations in each municipality for recruitment to the TMM CommCohort study, (2) in obstetric clinics and hospitals for the recruitment of pregnant women and their families and recording of clinical data for the TMM BirThree cohort, and (3) in seven Community Support Centers in Miyagi Prefecture for physiological measurements in both studies.

Assistants are employed to bridge the gap between participants and researchers, with informed consent (IC) obtained via face-to-face explanation and in a written format according to the Japanese Ethical Guidelines for Human Genome/Gene Analysis Research [5]. Based on these guidelines, the Japanese Society of Human Genetics (JSHG) established a certificated education system in FY 2008 to train assistants in the IC procedure (ICP). These assistants are referred to as genome medical research coordinators (GMRCs) [6]. In FY 2012, the TMM proposal issued via the Japanese Ministry of Education, Culture, Sports, Science and Technology (MEXT) highlighted the importance of considering the ethical issues related to obtaining consent from participants via appropriate face-to-face explanation by educated staff [7]. Subsequently, in the plan of action, it was estimated that more than 150 GMRCs would be required by the ToMMo during the recruitment and baseline assessments [8].

**Fig. 1:**
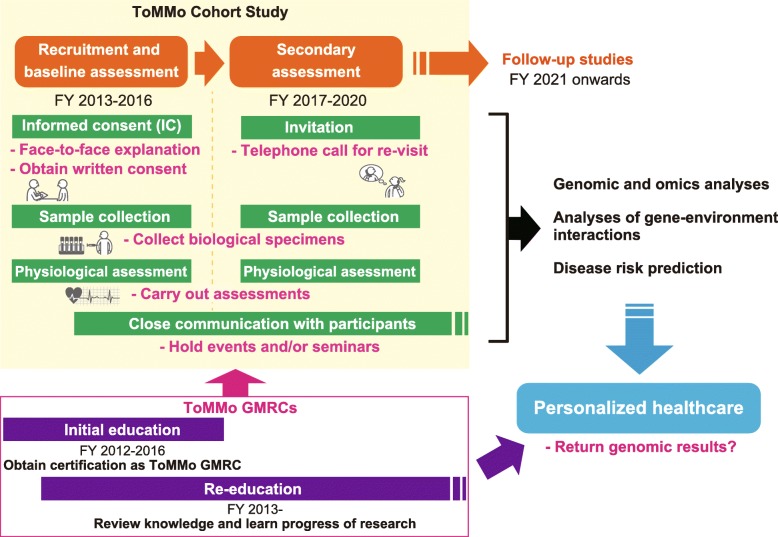
Roles of GMRCs during the large-scale cohort study of the ToMMo. For the ToMMo, 120,000 of 150,000 participants enrolled in the TMM study were recruited via face-to-face consent and baseline assessments were carried out in FY 2013–2016. A secondary assessment, “TMM Repeat Assessment Center-based Survey during the Second Period”, was then commenced in FY 2017, to be continued until FY 2020. Requirements of each stage of the study are shown in green, while essential roles of the ToMMo GMRCs are indicated below in magenta

We therefore decided to employ additional assistants living in Miyagi Prefecture to work specifically as ToMMo GMRCs. In addition to the abovementioned roles, these individuals were also expected to be responsible for continuous communication with participants throughout the long-term study, thereby helping maintain a high rate of follow-up (Fig. 1). Close communication is particularly important in building a trusting relationship with participants, thereby helping maintain the motivation required to remain in a long-term study. We therefore organized a ToMMo GMRC committee ran by ToMMo researchers with the aim of developing an education and training program for ToMMo GMRCs, surpassing that of JSHG GMRCs. To ensure a certain level of knowledge and skills to carry out the ICP and other practical roles, the program covers the knowledge required by JSHG GMRCs as well as providing an understanding of the TMM study and the ICP (for details see Additional file 1: Table S1). ToMMo GMRC certification is awarded by attending lectures and a practical training session followed by written and interview examinations. After qualification, ToMMo GMRCs are then able to carry out ICP at recruitment sites. In addition, on-the-job training is also conducted to teach the skills required for biological sample collection and physiological measurements. A total of 296 ToMMo GMRCs were certified during the recruitment and baseline assessment period. Of these, 183 also obtained JSHG GMRC certification prior to FY 2016 (a total of 533 GMRCs were certified by the JSHG by FY 2016). In addition, these ToMMo GMRCs also attended workshops to keep them updated on each cohort study and any developments in genomic research.

Individual studies within large-scale cohort studies and/or biobank projects use different types of personnel to obtain IC and/or conduct assessments. For example, some studies use general practitioners to recruit participants and have research nurses perform the examinations [9]. In others, obstetricians and midwives provide initial information and trained staff carry out ICP [10]. However, in most of these projects, it is unknown whether assistants undergo a specific training program. In contrast, the UK Biobank project created and published a core training program for all members of staff in their assessment centers during the recruitment of half a million participants from 2006 to 2010 [11, 12]. This training program was conducted by UK Biobank’s clinical operation manager for 3–5 days prior to opening the assessment centers, and consisted of five sessions. JSHG GMRC certification also involves five lectures covering epidemiology, human genetics, database use, research ethics, and communication skills followed by a written examination. Practical training is not a definitive requirement and IC skills are not checked during the exam. A comparison of the education and training programs carried out by UK Biobank and that created for JSHG and ToMMo GMRCs is shown in Table 1. The ToMMo GMRC program incorporates lectures covering basic knowledge, as implemented by the JSHG, on-the-job training, as in the UK Biobank program, and practical training in the form of ICP role-playing.

**Table 1:** Comparison of education and training programs for assistants who support cohort studies and/or biobank projects

	Core training program of UK Biobank (2006–2010) [8]	Certification system for JSHG GMRCs (FY2008-) [4]	Education and training program for ToMMo GMRCs (FY 2012-)
Initial education			
Period	3–5 days	1 day	Approximately 4 weeks
Program	5 sessions	5 lectures	18 sessions
Lectures	1 session: project overview, consent process etc. 3 sessions: practice (questionnaire, physical measurements, sample collection and processing)	5 lectures: epidemiology, human genetics, databasing, research ethics, and communication skills	8 lectures: epidemiology, human genetics etc. 9 lectures: background to the TMM study, and ToMMo practices
Practical training	1 session: includes practical experience of a baseline assessment visit	None	1 session: 2-h role-play training in the informed consent procedure
Examinations	No information	Written examination	Written and interview examinations
Other	On-the-job training	None	On-the-job training
Re-education			
Program	No information	Advanced seminar and other seminars authorized by JSHG	Annual re-education workshop and small group re-education workshops
Advanced certification	No information	Senior GMRC certification (IC experience and credits gained from seminar attendance)	Advanced ToMMo GMRC (IC experience and credits gained from seminar attendance)
Renewal	No information	Once every 5 years	Once every 5 years

*GMRC* genome medical research coordinator, *JSHG* Japanese Society of Human Genetics, *ToMMo* Tohoku Medical Megabank Organization, *TMM* Tohoku Medical Megabank Project

To our knowledge, no report has yet documented education programs aimed specifically at assistant staff in cohort and/or biobank projects. However, understanding the effectiveness of these programs is important for training ToMMo GMRCs. In this study, we therefore examined the overall level of understanding, usefulness, and satisfaction of various lectures and training programs via a comprehensive questionnaire survey of qualified ToMMo GMRCs.

## Methods

### Education and training program for ToMMo GMRCs

The education program for ToMMo GMRCs was divided into two parts: initial education and training, and re-education. ToMMo staff who conduct ICP are required to acquire ToMMo GMRC certification via an initial education and training program consisting of 18 sessions and 2 examinations. These initial 18 sessions are composed of 17 1-h lectures and a 2-h practical ICP training sessions (for details see Additional file 1: Table S1). Live lectures are carried out by ToMMo researchers in the education and training division as well as other divisions related to cohorts, biobanks, information and communications technology, and so on. DVD recorded lectures are also used when there are few attendees. ICP training is carried out by a pair of trainees in the form of recruitment scene role-play, after which two types of examination are held: a written examination to judge basic knowledge of the ICP, and an interview examination to evaluate skills in implementing the ICP. The latter also takes the form of a role-play, with simulated candidate participants. The evaluation is performed according to 8 criteria (Additional file 2: Text 1). Certification is then awarded to those who pass both examinations. The period of implementation of the initial program is around 4 weeks.

The re-education program consists of two types of workshop: an obligatory annual re-education workshop covering any progress that has been made in the cohort studies as well as other important topics involved in activities of the ToMMo, and voluntary small group workshops held several times a year and dealing with topics related to the TMM study and genomic research. The latter workshops are carried out in the form of lectures, discussions, and training. Since it is not easy to gather all ToMMo GMRCs in one venue for the small group re-education workshops, a video conferencing system was used to allow participants from each community support center to join in real time. Moreover, the Internet School of Tohoku University (ISTU) distributed streaming video recordings of each workshop, thereby supporting updates at any time. Through participation in these workshops and related seminars, GMRCs can apply for credits, which lead to the advanced ToMMo GMRC certification and certification renewal, which is required every 5 years.

### Questionnaire survey

A questionnaire was designed to specifically evaluate the education and training program for ToMMo GMRCs. As shown in Additional file 3: Text 2, the questionnaire consisted of six parts: (1) demographic characteristics, (2) evaluation of the initial education and training program, (3) evaluation of the small group re-education workshops, (4) evaluation of the annual re-education workshop, (5) evaluation of overall knowledge of epidemiology, genetics, and genomic medicine, and (6) an open comments section. This was a one-off survey, with anonymous questionnaires sent along with a written explanation to 152 qualified ToMMo GMRCs working in the ToMMo in February 2017, all of whom were close to completing the recruitment and initial education and training program. Responses were received within 1 month by postal mail to maintain anonymity. The study was approved by the Research Ethics Committee of Tohoku Medical Megabank Organization, Tohoku University, Japan.

### Statistical analysis

All data analyses were performed using SAS Enterprise Guide 7.11 (SAS Institute Inc.). Evaluation of lectures was compared with that of practical training using Fisher’s exact test. Associations between evaluations and the demographic characteristics were examined using logistic regression analysis. The overall understanding of lectures related to basic knowledge of genetics and epidemiology were compared with that of those related to ToMMo GMRC practices using the χ^2^-test. The associations between levels of understanding and the usefulness of each lesson were examined using Pearson’s correlation analysis. Lastly, the rate of selection of particular re-education topics was compared with that of all other topics using Fisher’s exact test.

## Results

### Demographic characteristics

To evaluate the education and training program, 152 qualified ToMMo GMRCs working in the organization as of February 2017 were enrolled in the study. Of these, 94 (61.8%) returned the questionnaire. Demographic characteristics of the respondents are shown in Table 2. More than 95% were women in their 30s, 40s, or 50s with various educational backgrounds. Almost half did not have any medical qualifications in nursing or as a clinical laboratory technologist. Nearly 90% obtained JSHG GMRC certification. The number of ICPs carried out varied from 100 to 600 depending on when they commenced and on their job content during the recruitment period, which ranged from obtaining IC, collecting biological specimens, recording clinical data, and/or carrying out physiological assessments.

**Table 2:** Demographic characteristics of the ToMMo GMRCs who responded to the questionnaire

	Survey respondents (n = 94)
Sex	n (%)
Female	90 (95.7)
Male	3 (3.2)
No answer	1 (1.1)
Age (years)	
< 29	4 (4.3)
30–39	26 (27.7)
40–49	42 (44.7)
50–59	22 (23.4)
Education	
High school	21 (22.3)
Vocational school	40 (42.6)
Junior college	9 (9.6)
University	17 (18.1)
Graduate school	5 (5.3)
Other	2 (2.1)
Medical qualification	
None	47 (49.5)
Nurse	34 (35.8)
Clinical laboratory technologist	7 (7.4)
Medical clerk	4 (4.2)
Other	2 (2.1)
No answer	1 (1.1)
Qualified as a ToMMo GMRC in	
FY 2013	52 (55.3)
FY 2014	35 (37.2)
FY 2015	3 (3.2)
FY 2016	3 (3.2)
No answer	1 (1.1)
JSHG GMRC certification	
Yes	81 (86.2)
No	12 (12.8)
No answer	1 (1.1)
Previous study involvement	
TMM CommCohort	16 (17.0)
TMM BirThree Cohort	49 (52.1)
Both	26 (27.7)
Other	3 (3.2)
Number of ICPs carried out	
0–10	2 (2.1)
11–50	12 (12.8)
51–100	6 (6.4)
101–200	15 (16.0)
201–400	22 (23.4)
401–600	21 (22.3)
> 600	14 (14.9)
No answer	2 (2.1)

*ToMMo GMRC* Tohoku Medical Megabank Organization genome medical research coordinator, *JSHG GMRC* Japanese Society of Human Genetics genome medical research coordinator, *TMM* Tohoku Medical Megabank Project, *ICP* informed consent procedure

### Evaluation of the initial education and training program

The initial education and training program composed of lectures and a practical training session (ICP role-play) was evaluated in terms of the time taken, level of difficulty, confidence gained, and overall satisfaction (Table 3). More than half of the respondents indicated that the time spent on both lectures and practical training was appropriate, and that both gave them confidence and an overall level of satisfaction. In addition, time taken and the level of difficulty significantly differed between lectures and practical training (*p* = 0.0009 and *p* < 0.0001, respectively), with lectures considered harder and more time consuming.

**Table 3:**
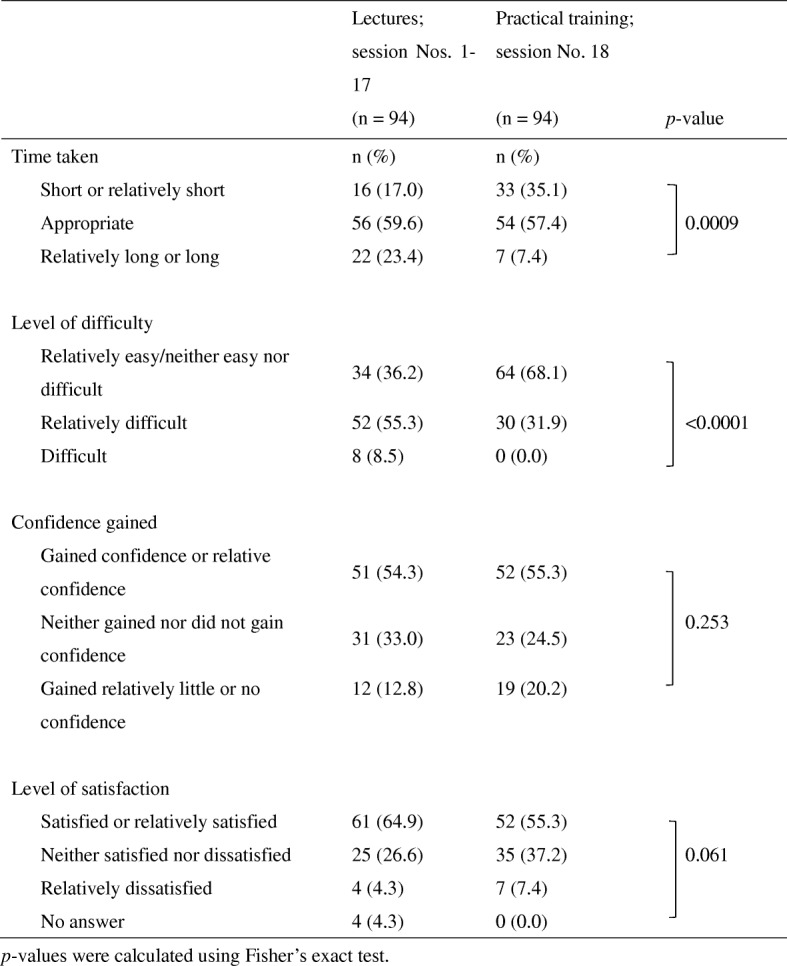
Evaluation of the initial education and training program

*p*-values were calculated using Fisher’s exact test

We subsequently analyzed whether these evaluations were correlated with the demographic characteristics of the ToMMo GMRCs. For this purpose, age, educational background, and medical qualifications were respectively categorized into two groups: < 40 or ≥ 40 years of age, university/graduate school degree or others, and nurse/clinical laboratory technologist or other (for details see Additional file 4: Table S2 and Additional file 5: Table S3). Associations between each demographic characteristic and the evaluations of lectures and practical training were then examined by logistic regression analysis. As shown in Tables 4 and 5, no associations were observed except for the level of difficulty of the lectures, which was significantly correlated with medical qualifications in nursing or as a clinical laboratory technologist (Odds ratio 0.33, *p* = 0.016).

**Table 4:** Association between evaluation of the 17 lectures and the participants’ demographics

	n (%)		Odds ratio (95% CI)	*p*-value
Time taken (*n* = 93)^e^	Short^a^	Others		
Age				
< 40	5 (5.4)	24 (25.8)	1.0 (ref)	–
≥ 40	11 (11.8)	53 (57.0)	0.74 (0.22–2.51)	0.630
Educational background				
University/graduate school	1 (1.1)	20 (21.5)	1.0 (ref)	–
Others	15 (16.1)	57 (61.3)	5.79 (0.69–48.51)	0.105
Medical qualification				
Nurse/clinical laboratory technologist	7 (7.5)	34 (36.6)	1.0 (ref)	–
Others	9 (9.7)	43 (46.2)	1.12 (0.37–3.41)	0.842
Level of difficulty (n = 93)^e^	Easy^b^	Others		
Age				
< 40	10 (10.8)	19 (20.4)	1.0 (ref)	–
≥ 40	23 (24.7)	41 (44.1)	1.31 (0.48–3.53)	0.598
Educational background				
University/graduate school	8 (8.6)	13 (14.0)	1.0 (ref)	–
Others	25 (27.9)	47 (50.5)	0.73 (0.25–2.14)	0.563
Medical qualification				
Nurse/clinical laboratory technologist	20 (21.5)	21 (22.6)	1.0 (ref)	–
Others	13 (14.0)	39 (41.9)	0.33 (0.14–0.81)	0.016
Confidence gained (n = 93)^e^	Gained^c^	Others		
Age				
< 40	16 (17.2)	13 (14.0)	1.0 (ref)	–
≥ 40	34 (36.6)	30 (32.3)	0.96 (0.38–2.39)	0.921
Educational background				
University/graduate school	12 (12.9)	9 (9.7)	1.0 (ref)	–
Others	38 (40.9)	34 (36.6)	0.85 (0.31–2.35)	0.752
Medical qualification				
Nurse/clinical laboratory technologist	22 (23.7)	19 (20.4)	1.0 (ref)	–
Others	28 (30.1)	24 (25.8)	1.00 (0.44–2.30)	0.994
Level of Satisfaction (*n* = 89)^f^	High^d^	Others		
Age				
< 40	20 (22.5)	9 (10.1)	1.0 (ref)	–
≥ 40	40 (44.9)	20 (22.5)	0.98 (0.36–2.64)	0.964
Educational background				
University/graduate school	14 (15.7)	6 (6.7)	1.0 (ref)	–
Others	46 (51.7)	23 (25.8)	0.84 (0.27–2.58)	0.759
Medical qualification				
Nurse/clinical laboratory technologist	29 (32.6)	11 (12.4)	1.0 (ref)	–
Others	31 (34.8)	18 (20.2)	0.65 (0.26–1.62)	0.354

^a^Short/relatively short

^b^Easy/relatively easy/neither easy nor difficult

^c^Gained confidence/gained some confidence

^d^Satisfied/relatively satisfied

^e^Removed 1 subject with missing value in medical qualification

^f^Removed 1 and 4 subject with missing value in medical qualification and in level of satisfaction, respectively

**Table 5:** Association between evaluation of the practical training session and the participants’ demographics

	n (%)		Odds ratio (95% CI)	*p*-value
Time taken (n = 93)^e^	Short^a^	Others		
Age				
< 40	10 (10.8)	19 (20.4)	1.0 (ref)	–
≥ 40	23 (24.7)	41 (44.1)	0.99 (0.38–2.64)	0.991
Educational background				
University/graduate school	5 (5.4)	16 (17.2)	1.0 (ref)	–
Others	28 (30.1)	44 (47.3)	1.97 (0.62–6.26)	0.251
Medical qualification				
Nurse/clinical laboratory technologist	18 (19.4)	23 (24.7)	1.0 (ref)	–
Others	15 (16.3)	37 (39.8)	0.53 (0.22–1.27)	0.156
Level of difficulty (n = 93)^e^	Easy^b^	Others		
Age				
< 40	19 (20.4)	10 (10.8)	1.0 (ref)	–
≥ 40	44 (47.3)	20 (21.5)	1.49 (0.56–4.02)	0.427
Educational background				
University/graduate school	17 (18.3)	4 (4.3)	1.0 (ref)	–
Others	46 (49.5)	26 (28.0)	0.36 (0.10–1.24)	0.106
Medical qualification				
Nurse/clinical laboratory technologist	29 (31.2)	12 (12.9)	1.0 (ref)	–
Others	34 (36.6)	18 (19.4)	0.71 (0.29–1.76)	0.458
Confidence gained (n = 93)^e^	Gained^c^	Others		
Age				
< 40	17 (18.3)	12 (12.9)	1.0 (ref)	–
≥ 40	34 (36.6)	30 (32.3)	0.83 (0.33–2.10)	0.692
Educational background				
University/graduate school	13 (14.0)	8 (8.6)	1.0 (ref)	–
Others	38 (40.9)	34 (36.6)	0.74 (0.26–2.07)	0.563
Medical qualification				
Nurse/clinical laboratory technologist	21 (22.6)	20 (21.5)	1.0 (ref)	–
Others	30 (32.3)	22 (23.7)	1.30 (0.57–3.00)	0.534
Level of Satisfaction (n = 93)^e^	High^d^	Others		
Age				
< 40	17 (18.3)	12 (12.9)	1.0 (ref)	–
≥ 40	34 (36.6)	30 (32.3)	0.76 (0.30–1.92)	0.561
Educational background				
University/graduate school	12 (12.9)	9 (9.7)	1.0 (ref)	–
Others	39 (41.9)	33 (35.5)	0.99 (0.36–2.75)	0.984
Medical qualification				
Nurse/clinical laboratory technologist	20 (21.5)	21 (22.6)	1.0 (ref)	–
Others	31 (33.3)	21 (22.6)	1.59 (0.69–3.68)	0.275

^a^Short/relatively short

^b^Easy/relatively easy/neither easy nor difficult

^c^Gained confidence/gained some confidence

^d^Satisfied/relatively satisfied

^e^Removed 1 subject with missing value in medical qualification

**Fig. 2:**
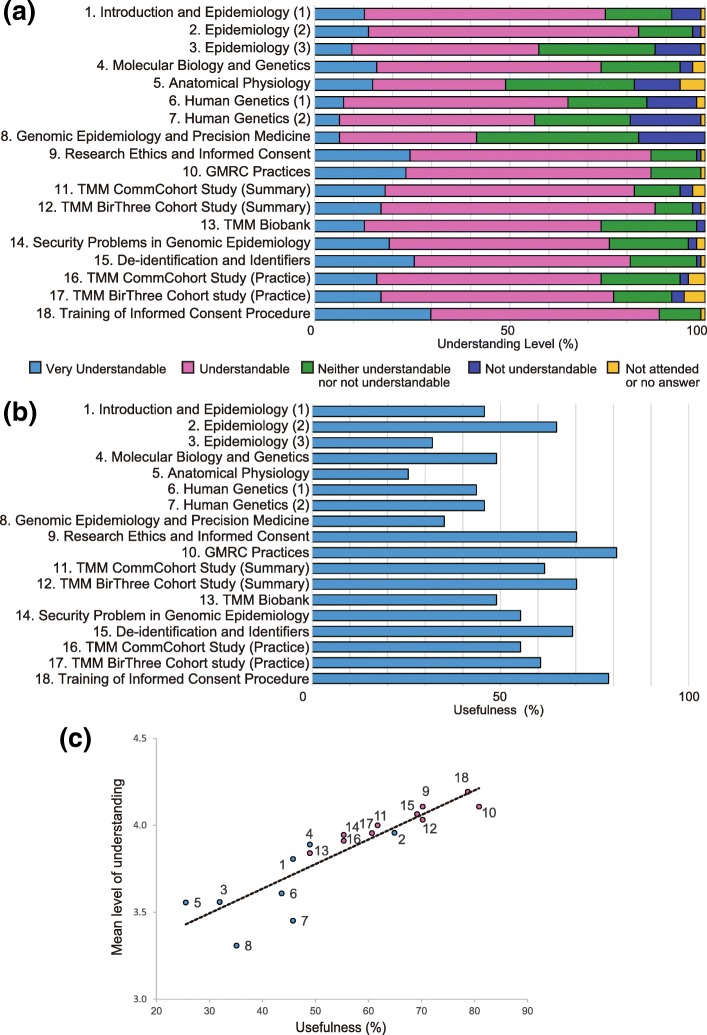
Relationship between overall understanding and usefulness of the ToMMo GMRC education and training program. The 18 sessions were composed of 17 lectures (Nos. 1 to 17) and one practical training exercise (No. 18), as indicated in the figure. **a** Level of understanding of each session on a 5-point Likert scale. **b** Percentage of respondents who found each lesson useful in their job. **c** Association between the level of understanding and overall usefulness of each session. The levels of understanding shown in (**a**) were converted into numerical values from 1 to 5 according to a low to high level of understanding then the mean values were calculated. Session Nos. 1–8, categorized as basic knowledge of genetics and epidemiology, are shown in blue, while session Nos. 9–18, categorized as GMRC practices, are shown in magenta. Pearson’s correlation coefficient was 0.89 (*p* < 0.0001)

Next, the overall level of understanding and the usefulness of each session were examined using a 5-point Likert scale. As shown in Fig. 2a, more than 80% of respondents found session Nos. 2 (Epidemiology (2)), 9 (Research Ethics and Informed Consent), 10 (GMRC Practices), 11 (TMM CommCohort Study (Summary)), 12 (TMM BirThree Cohort Study (Summary)), 15 (De-identification and Identifiers), and 18 (Practical Training on the ICP) understandable. Each of these sessions, except for No. 2, which covered the basics of observational studies in epidemiology, were related to ToMMo GMRC practices. Meanwhile, the following sessions were deemed neither understandable nor not understandable by more than 40% of respondents: session Nos. 3 (Epidemiology (3)), 5 (Anatomical Physiology), 7 (Human Genetics (2)), and 8 (Genomic Epidemiology and Precision Medicine), all of which are related to basic knowledge of genetics and epidemiology. In line with this, the rate of very understandable to understandable was lowest in terms of session No. 8 (41% of respondents), suggesting particular difficulty in understanding recent advances in genomic medicine. Moreover, when the 18 sessions were roughly divided into two groups, ToMMo GMRC practices (session Nos. 9 to 18) and overall knowledge of genetics and epidemiology (session Nos. 1 to 8), the overall level of understanding was significantly higher with the former (Table 6, *p* < 0.0001).

**Table 6:** Overall understanding of lectures related to genetics and epidemiology and ToMMo GMRC practice, respectively

	Category		
	Basic knowledge of genetics and epidemiology; session Nos. 1–8 (*n* = 737)	ToMMo GMRC practices; session Nos. 9–18 (*n* = 921)	*p*-value
Understanding	n (%)	n (%)	
Understandable^a^	469 (63.6)	761 (82.6)	< 0.0001
Not understandable^b^	268 (36.4)	160 (17.4)	

*ToMMo GMRC* Tohoku Medical Megabank Organization genome medical research coordinator

^a^Very understandable/understandable

^b^Neither understandable nor not understandable/not understandable

n indicates the total numbers of answers against each session without missing values

*p*-value was calculated using the χ^2^-test

Meanwhile, the rate of usefulness was 70% or more for session Nos. 9, 10, 12, 15, and 18, all of which are related to ToMMo GMRC practices (Fig. 2b). Furthermore, a strong correlation was observed between the overall level of understanding and rate of usefulness (Fig. 2c, *p* < 0.0001). These results suggest that ToMMo GMRCs clearly understand topics directly related to their work, but are less knowledgeable on epidemiology, human genetics, and genomic medicine, which form the background to the TMM study.

### Evaluation of the re-education program

**Fig. 3:**
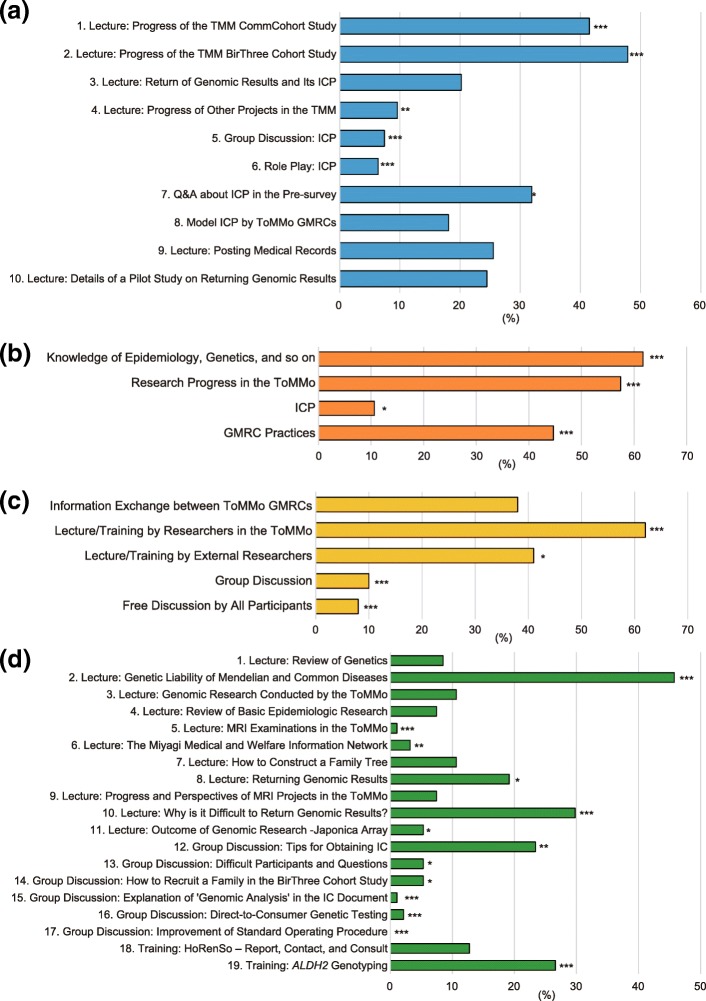
Evaluation and expectations of the (**a**-**c**) annual re-education workshop and (**d**) small group re-education workshops. **a**, **d** Topics currently covered and the overall preference of the ToMMo GMRCs. **b** Topics and (**c**) styles of learning preferred in future workshops. All questions allowed multiple answers (up to 3 in (**a**) and (**d**)). Rates of selection are indicated (*n* = 94). *** *p* < 0.001, ** *p* < 0.01, * *p* < 0.05 vs. the mean rate of selection of all other contents according to Fisher’s exact test

In addition to the initial education program, a re-education program was also developed to review knowledge of genetics and epidemiology and provide an update on progress in the TMM study and any cutting edge advances in genomic research and medicine. In the annual re-education workshop, more than 40% of respondents preferred the lectures on the progress of cohort studies (nos. 1 and 2) followed by questions and answers about the ICP in the pre-survey (No. 7) (Fig. 3a). Furthermore, in the future, they expressed a desire to learn more about epidemiology and genetics, research progress in ToMMo, and GMRC practices, as taught by both internal and external researchers (Fig. 3b and c).

Meanwhile, in the small group re-education workshops, lectures on the genetic liability of Mendelian and common diseases (No. 2) and the return of genomic results (returning individual genomic results of the participants) (Nos. 8 and 10) were the most selected topics (Fig. 3d). In addition, group discussions on the ICP (No. 12) and training of *ALDH2* genotyping (No. 19) were also favored. These results suggest that ToMMo GMRCs are interested in learning both about GMRC practices, which are directly useful in their job, and knowledge relevant to the background, results, and perspectives of TMM studies, all of which help with self-improvement. Note that while 60% of participants participated in the re-education program via alternative forms of education such as video conferencing and online workshops, comparisons of online with face-to-face participation revealed that the rate of understanding was nearly the same (about 80%), while the rate of satisfaction was lower with online workshops (58% vs. 84%, data not shown).

### Current knowledge among ToMMo GMRCs

**Fig. 4:**
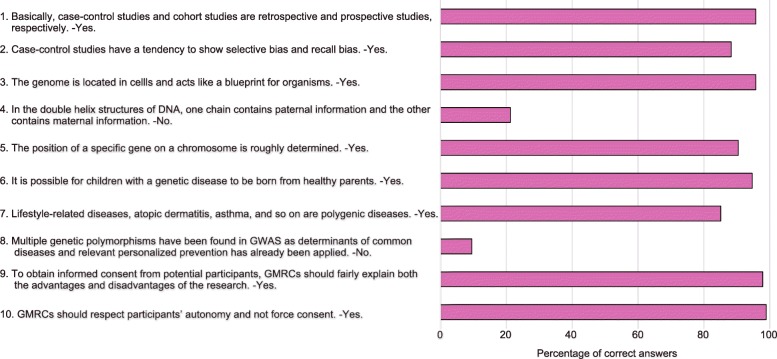
Percentage of correct answers in the 10-question quiz (*n* = 94). Each question and correct answer are indicated

Lastly, we examined the level of current knowledge among ToMMo GMRCs using a questionnaire quiz consisting of 10 questions conducted on February 2017. The percentage of correct answers is shown in Fig. 4. Rates of correct answers to session Nos. 4 and 8, which are related to basic genetics and human genetics, respectively, were extremely low, suggesting that ToMMo GMRCs failed to acquire this knowledge during the initial education and training program (Fig. 2a).

## Discussion

In this study, we described and evaluated the education and training program developed especially for ToMMo GMRCs. As a result, we found that while the program increases confidence, participants face difficulties understanding basic genomic medicine. Continuous re-education is therefore extremely important in increasing knowledge and skills. To the best of our knowledge, the trial described here is the first example of a systematic educational program specifically for personnel responsible for communicating with participants in a genomic epidemiological study. The findings could therefore be applied to similar human resource training programs in other studies.

The education and training program for ToMMo GMRCs was developed to support establishment of large cohorts. In the initial education and training program, lectures not only cover the knowledge required by JSHG GMRCs, but were extended to deliver more extensive knowledge via seven lectures covering topics ranging from epidemiology to human genetics. Furthermore, efforts of the ToMMo were also incorporated into these lectures, such as informational security, de-identification, and bio-banking. Evaluation of the ToMMo GMRC program revealed that participants deem the lectures more difficult and more time consuming than the practical training, suggesting that the lectures were a little too hard and of less overall use in carrying out the ICP. However, we believe that lectures indirectly related to the skills required for the ICP are important in understanding and being able to explain the significance of the TMM project to participants. The level of difficulty of the lectures was also significantly correlated with a lack of medical qualifications, suggesting that some lectures, such as the fundamentals of epidemiology and genetics, can be omitted for those with a medical background or qualification and for those with a sufficient level of knowledge as confirmed by a preliminary test. Taken together, the findings of this study suggest that the initial education and training program for ToMMo GMRCs is a unified program applicable across a diverse demographic range, ultimately providing a certain skill level for implementation of the ICP. Moreover, by having an overall understanding of the background and purpose of the TMM, cooperation among ToMMo GMRCs should be possible, thereby increasing overall motivation. For this reason, it is important to compensate for the lack of knowledge, especially in recent advances in genomic medicine, via continuous re-education.

The two types of re-education workshops were deemed particularly useful not only in reviewing what was learned in the initial program, but also in keeping participants up to date on any progress in the field. Responses to the questionnaire further suggested that these workshops are also an important opportunity for ToMMo GMRCs to make contact with researchers, thereby increasing motivation. A preference was shown for lectures dealing with the progress in cohort studies and topics related to human genetics and epidemiology, such as the causes of common diseases, thereby supporting what was previously learned but not yet fully understood. Re-education via video conferencing and online resources received a high rate of understanding but a lower rate of satisfaction, suggesting that alternative forms of re-education are of use to only a certain degree. By analyzing completers of a massive open online course (MOOC) recently developed to support online and open access learning opportunities, it was found that having an understanding of participants’ motivation factors, which range from improving knowledge, obtaining certification, and professional advancement, is crucial for the development of MOOCs [13]. Providing as many opportunities as possible for re-education and self-improvement of ToMMo GMRCs is therefore important, followed by evaluation of the most effective methods. In line with this, based on the results of our quiz, basic knowledge of human genetics was consistently low following the initial education period, suggesting that constant re-education is of vital importance.

There are two limitations of this study. One is selection bias of respondents due to the response rate of only 60%. The other is recall bias as more than 90% of ToMMo GMRCs participated in the initial education and training program in FY 2013 or 2014 (Table 2), but didn’t carry out the questionnaire until FY 2016.

To summarize, in our TMM study, we emphasized the importance of face-to-face ICP in order to improve overall understanding among participants. Some studies use electronic consent, with no face-to-face assistance [14, 15]. A recent prospective randomized study showed that the use of multimedia modules such as slide presentations with narrative tools take more time to complete, but improve the overall understanding of participants compared with face-to-face explanation [16]. This finding suggests that the use of multimedia during the ICP is efficient in certain projects requiring one off consent and collection of biological samples; however, to build a relationship of trust for long-term follow-up studies like the TMM study, face-to-face communication is thought to be more beneficial. We are now conducting secondary assessments of our participants via physiological testing in seven community support centers. ToMMo GMRCs are not only expected to collect physical/blood data, but are also required to contact participants for re-visits and talk with participants in order to diminish anxiety during assessment. In a previous study, it was shown that participants with low overall motivation or motivation fueled by money/gifts tend to have more concerns and a poorer understanding of a long-term cohort study, potentially increasing the risk of dropout [17]. It was also reported that the experience of consenters affected the willingness of patients enrolled in a university-based cancer center tissue repository during follow-up and future research [18]. Communication between experienced ToMMo GMRCs and participants is therefore very important in maintaining or even increasing participant cooperation. The abovementioned lectures, which aimed at understanding the significance of the TMM project, would be useful for maintaining communication.

As the framework of medical care, education of medical staff is important. For example, the Genomics Education Program in Genomics England provides various education courses and tools supported by Health Education England [19, 20]. We were unable to perform medical care within the research framework of the TMM study; however, we aim to translate the results of the TMM study for wider genomic medicine research. Thus, by receiving training on clinical genetics, ToMMo GMRCs could be in charge of returning genomic results to participants [21]. That is, in this way, we hope that ToMMo GMRCs could become specialists in community-based healthcare. Taken together, the findings of this study suggest that ToMMo GMRCs found it difficult to understand the genetic aspects of the initial education and training program, highlighting the importance of a continuous re-education program aimed at keeping participants up to date on the latest progress and increasing specialist knowledge.

## Conclusions

The education and training program for ToMMo GMRCs was developed out of a necessity for assistants able to provide vital support in large-scale cohort studies such as the ToMMo. Such assistants are required to obtain IC, implement assessments, and communicate with participants. Evaluation of the program by nearly 100 ToMMo GMRCs suggests that the initial program increased confidence and provided overall satisfaction to more than half of participants; however, knowledge on genetics and epidemiology, especially in terms of recent progress in genomic medicine, remains difficult. Continuous re-education is therefore vital for self-improvement and career development. In conclusion, this study evaluated, for the first time, an education and training program aimed specifically at assistants in a genome cohort study in Japan. The program was shown to be useful, resulting in the acquisition of basic and practical knowledge, and is also thought to be applicable to human resource development in other large-scale cohort studies.

## Additional files

Additional file 1: **Table S1.** The 18 sessions comprising the initial education and training program for ToMMo GMRCs. (DOCX 19 kb)

Additional file 2: **Text 1.** Evaluation criteria of the ToMMo GMRC interview examination. (DOCX 19 kb)

Additional file 3: **Text 2.** Questionnaire items used to evaluate the education and training program for ToMMo GMRCs. (DOCX 53 kb)

Additional file 4: **Table S2.** Evaluation of the 17 lectures in the initial education and training program according to the participants’ demographics. (DOCX 25 kb)

Additional file 5: **Table S3.** Evaluation of the practical training session in the initial education and training programs according to participants’ demographics. (DOCX 19 kb)

## Data Availability

The datasets used for this study are available from the corresponding authors on reasonable request.

## Abbreviations

FY: Fiscal year
GMRC: Genome medical research coordinator
IC: Informed consent
ICP: Informed consent procedure
JSHG: Japanese Society of Human Genetics
MOOC: Massive open online course
TMM BirThree Cohort: TMM Birth and Three-Generation Cohort Study
TMM CommCohort: TMM Community-Based Cohort Study
TMM: Tohoku Medical Megabank Project
ToMMo: Tohoku Medical Megabank Organization
