# Supplementary material for: A training and education program for genome medical research coordinators in the genome cohort study of the Tohoku Medical Megabank Organization
Source: BMC Med Educ. 2019 Aug 2;19:297. doi: 10.1186/s12909-019-1725-5 (PMC6679441; doi:10.1186/s12909-019-1725-5)
Supplement: Supplementary file 1 — Table S1. The 18 sessions comprising the initial education and training program for ToMMo GMRCs. (DOCX 19 kb) [file 12909_2019_1725_MOESM1_ESM.docx]

**Additional Table 1 The 18 sessions comprising the initial education and training program for Tohoku Medical Megabank Organization genome medical research coordinators (ToMMo GMRCs)**

| **No.** | **Name of session**^a^ | **Main content** |
| --- | --- | --- |
| 1 | Introduction, Epidemiology (1) | What is a GMRC?  Prevalence/incidence rate |
| 2 | Epidemiology (2) | Case-control/Cohort studies  Research design and bias |
| 3 | Epidemiology (3) | Sensitivity/specificity of inspection  Positive/negative predictive values |
| 4 | Molecular Biology and Genetics | Genomes/chromosomes/genes/DNA  Central dogma |
| 5 | Anatomical Physiology | Organization of the human body |
| 6 | Human Genetics (1) | Monogenic diseases  DNA mutations and polymorphisms |
| 7 | Human Genetics (2) | Polygenic diseases  Quantitative and qualitative traits |
| 8 | Genomic Epidemiology and Precision Medicine | Genome-wide association studies (GWAS)  Next-generation sequencing (NGS) |
| 9 | Research Ethics and Informed Consent | History of medical research  Ethical guidelines in Japan |
| 10 | GMRC Practices | Work of GMRCs  Interview techniques |
| 11 | TMM CommCohort Study (Summary) | Study purpose and strategy |
| 12 | TMM BirThree Cohort Study (Summary) | Study purpose and strategy |
| 13 | TMM Biobank | Organization of the TMM Biobank  Handling of biological samples |
| 14 | Security Problems in Genomic Epidemiology | Privacy protection  Information management |
| 15 | De-identification and Identifiers | Identifiers used in the TMM |
| 16 | TMM CommCohort Study (Practice) | Study procedures |
| 17 | TMM BirThree Cohort study (Practice) | Study procedures |
| 18 | Practical Training on the Informed Consent Procedure | GMRC role play |

TMM: Tohoku Medical Megabank Project.

^a^Session Nos. 1 to 17 consist of lectures and session No. 18 of practical training.

Of note, an additional session, Introduction of Genetic Counseling, is included from the middle of FY 2013.
