# Supplementary material for: A training and education program for genome medical research coordinators in the genome cohort study of the Tohoku Medical Megabank Organization
Source: BMC Med Educ. 2019 Aug 2;19:297. doi: 10.1186/s12909-019-1725-5 (PMC6679441; doi:10.1186/s12909-019-1725-5)
Supplement: Supplementary file 2 — Text 1. Evaluation criteria of the ToMMo GMRC interview examination. (DOCX 19 kb) [file 12909_2019_1725_MOESM2_ESM.docx]

**Additional Text 1 Evaluation criteria of the ToMMo GMRC** **interview examination**

(A. Distinction, B. Merit, C. Pass, D. Fail)

Evaluation:

1. Introduced him/herself.

A. B. C.

2. Sufficiently explained the ICP and time required.

A. B. C.

3. Spoke at an appropriate speed and with clear articulation.

A. B. C.

4. Provided accurate information.

　　 A. B. C.

5. Sufficiently explained the technical terms in a user-friendly manner.

A. B. C.

6. Took into account the facial expression and body language of the candidate participants.

A. B. C.

7. Took time to adequately answer any questions.

A. B. C.

8. Avoided unpleasant gestures and manners.

A. B. C.

Comprehensive evaluation:

A. B. C. D.
