# Supplementary material for: A training and education program for genome medical research coordinators in the genome cohort study of the Tohoku Medical Megabank Organization
Source: BMC Med Educ. 2019 Aug 2;19:297. doi: 10.1186/s12909-019-1725-5 (PMC6679441; doi:10.1186/s12909-019-1725-5)
Supplement: Supplementary file 3 — Text 2. Questionnaire items used to evaluate the education and training program for ToMMo GMRCs. (DOCX 53 kb) [file 12909_2019_1725_MOESM3_ESM.docx]

**Additional Text 2 Questionnaire items used to evaluate the education and training program for Tohoku Medical Megabank Organization Genome Medical Research Coordinators** **(ToMMo GMRCs)**

**Questionnaire on the ToMMo GMRC education and training program**

ToMMo Department of Education and Training

To help enhance the ToMMo GMRC education and training program, please complete the following questionnaire. *For further details, please refer to the instruction sheet provided.

[Question 1] Demographic characteristics

1. Sex

□ Female □ Male

2. Age (years)

□ 20s □ 30s □ 40s □ 50s □ 60s

3. Education

□ Junior high school □ High school □ Vocational school □ Junior college

□ University □ Graduate school □ Other ( )

4. Medical qualification

□ None □ Nurse □ Clinical laboratory technologist □ Other ( )

5. When started

□ FY 2013 □ FY 2014 □ FY 2015 □ FY 2016

6. JSHG GMRC certification

□ Yes □ No

7. Previous studies involved in

□ TMM CommCohort □ TMM BirThree Cohort □ Both □ Neither

8. Approximate number of informed consent procedures (ICP) conducted so far

□ 0-10 □ 11-50 □ 51-100 □ 101-200 □ 201-400 □ 401-600 □ >600

[Question 2] Evaluation of the initial education and training program

*Initial training prior to working as a GMRC

1. Lectures

| Time taken | □ Short □ Relatively short □ Appropriate □ Relatively long □ Long |
| --- | --- |
| Level of difficulty | □ Easy □ Relatively easy □ Neither easy nor difficult  □ Relatively difficult □ Difficult |
| Confidence gained | □ Gained confidence □ Gained some confidence  □ Neither gained nor did not gain confidence □ Gained little confidence □ Gained no confidence |
| Level of satisfaction | □ Satisfied □ Relatively satisfied □ Neither satisfied nor dissatisfied □ Relatively dissatisfied □ Dissatisfied |

2. Practical training

| Time taken | □ Short □ Relatively short □ Appropriate □ Relatively long □ Long |
| --- | --- |
| Level of difficulty | □ Easy □ Relatively easy □Neither easy nor difficult  □ Relatively difficult □ Difficult |
| Confidence gained | □ Gained confidence □ Gained some confidence  □ Neither gained nor did not gain confidence □ Gained little confidence □ Gained no confidence |
| Level of satisfaction | □ Satisfied □ Relatively satisfied □ Neither satisfied nor dissatisfied □ Relatively dissatisfied □ Dissatisfied |

3. Level of understanding at the time of each lecture and practical training session and the written examination. If you did not attend a specific session, please mark as “not attended”. In addition, please check the “usefulness” box if you found a session particularly useful in your job.

| Session | Level of understanding | | | | | Not attended | Usefulness |
| --- | --- | --- | --- | --- | --- | --- | --- |
|  | 1. Very understandable | 2. Understandable | 3. Neither understandable nor not understandable | 4. Relatively hard to understand | 5. Could not understand |  |  |
| Example | \|----------\|----------\|----------\|----------\| | | | | | □ | ☑ |
| 1. Introduction, Epidemiology (1)   (Prevalence/incidence rate, etc.) | \|----------\|----------\|----------\|----------\| | | | | | □ | □ |
| 1. Epidemiology (2) (Cohort study, etc.) | \|----------\|----------\|----------\|----------\| | | | | | □ | □ |
| 1. Epidemiology (3)   (Sensitivity/specificity of inspection, etc.) | \|----------\|----------\|----------\|----------\| | | | | | □ | □ |
| 1. Molecular Biology and Genetics   (Cell/DNA/protein, etc.) | \|----------\|----------\|----------\|----------\| | | | | | □ | □ |
| 1. Anatomical Physiology | \|----------\|----------\|----------\|----------\| | | | | | □ | □ |
| 1. Human Genetics (1) (Monogenic diseases, etc.) | \|----------\|----------\|----------\|----------\| | | | | | □ | □ |
| 1. Human Genetics (2)　(Polygenic diseases) | \|----------\|----------\|----------\|----------\| | | | | | □ | □ |
| 1. Genomic Epidemiology and Precision Medicine (GWAS/NGS, etc.) | \|----------\|----------\|----------\|----------\| | | | | | □ | □ |
| 1. Research Ethics and Informed Consent | \|----------\|----------\|----------\|----------\| | | | | | □ | □ |
| 10. GMRC Practices  　　(Interview technique) | \|----------\|----------\|----------\|----------\| | | | | | □ | □ |
| 11. TMM CommCohort Study (Summary) | \|----------\|----------\|----------\|----------\| | | | | | □ | □ |
| 12. TMM BirThree Cohort Study (Summary) | \|----------\|----------\|----------\|----------\| | | | | | □ | □ |
| 13. TMM Biobank | \|----------\|----------\|----------\|----------\| | | | | | □ | □ |
| 14. Security Problems in Genomic Epidemiology | \|----------\|----------\|----------\|----------\| | | | | | □ | □ |
| 15. De-identification and Identifiers | \|----------\|----------\|----------\|----------\| | | | | | □ | □ |
| 16. TMM CommCohort Study (Practice) | \|----------\|----------\|----------\|----------\| | | | | | □ | □ |
| 17. TMM BirThree Cohort Study (Practice) | \|----------\|----------\|----------\|----------\| | | | | | □ | □ |
| 18. Practical ICP Training | \|----------\|----------\|----------\|----------\| | | | | | □ | □ |

4. Any other comments on the initial education and training program.

[Question 3] Evaluation of the small group re-education workshops

1. Workshops

| Frequency of participation | □ All □ Almost all □ About half □ Some □ None |
| --- | --- |
| Appropriate frequency of workshops | □ Monthly □ Every 2 months □ Every 3 months  □ Every 4 months □ Twice yearly □ Other |
| Time taken per workshop | □ Short □ Relatively short □ Appropriate □ Relatively long □ Long |
| Level of understanding | □ Very understandable □ Understandable  □ Neither understandable nor not understandable  □ Not very understandable □ Not understandable |
| Usefulness | □ Useful □ Relatively useful □ Neither useful nor not useful  □ Not very useful □ Not useful |
| Level of satisfaction | □ Satisfied □ Relatively satisfied  □ Neither satisfied nor dissatisfied □ Relatively dissatisfied  □ Dissatisfied |

2. Use of TV conferencing and/or Internet School of Tohoku University (ISTU) streaming of workshops (levels of understanding and satisfaction compared with direct participation).

- Have you participated in a TV conference workshop?

□ Yes (Please complete the following) □ No

| Level of understanding | □ Very understandable □ Understandable  □ Neither understandable nor not understandable  □ Not very understandable □ Not understandable |
| --- | --- |
| Level of satisfaction | □ Satisfied □ Relatively satisfied  □ Neither satisfied nor dissatisfied □ Relatively dissatisfied  □ Dissatisfied |

- Have you used ISTU streamed workshops?

□ Yes (Please complete the following) □ No

| Level of understanding | □ Very understandable □ Understandable  □ Neither understandable nor not understandable  □ Not very understandable □ Not understandable |
| --- | --- |
| Level of satisfaction | □ Satisfied □ Relatively satisfied  □ Neither satisfied nor dissatisfied □ Relatively dissatisfied  □ Dissatisfied |

- Any other comments on TV conference and ISTU streamed workshops.

3. Please select a maximum of three sessions that you particularly enjoyed.

□ Lecture: Review of genetics

□ Lecture: Genetic liability of Mendelian and common diseases

□ Lecture: Genomic research conducted by the ToMMo

□ Lecture: Review of basic epidemiologic research

□ Lecture: MRI examinations in the ToMMo

□ Lecture: The Miyagi Medical and Welfare Information Network (MMWIN)

□ Lecture: How to construct a family tree

□ Lecture: Returning genomic results

□ Lecture: Progress and perspectives of MRI projects in the ToMMo

□ Lecture: Why is it difficult to return genomic results?

□ Lecture: Outcome of genomic research - Japonica array

□ Group discussion: Tips for obtaining IC

□ Group discussion: Difficult participants and questions

□ Group discussion: How to recruit a family in the BirThree cohort study

□ Group discussion: Explanation of 'Genomic analysis' in the IC document

□ Group discussion: Direct-to-consumer genetic testing

□ Group discussion: Improvements to standard operating procedure (SOP)

□ Training: HoRenSo – Report, Contact, and Consult

□ Experimental training: *ALDH2* genotyping

□ Other ( )

4. Please select which topics you hope to learn more about in future workshops. (No limit).

□ Epidemiology □ Genes and genomes □ Precision medicine

□ Research progress in the ToMMo □ Research ethics □Communication

□ Practical use □ Other ( )

5. Please select which style of learning you would prefer in future workshops (No limit).

□ Information exchange between ToMMo GMRCs

□ Lectures or training by internal researchers

□ Lectures or training by external researchers □ Free discussion

□ Group discussions □ Experimental training □ Other ( )

6. Evaluation of tasks submitted in FY 2014.

- Have you submitted any tasks?

□ Yes (Please complete the following) □ No

| Level of difficulty | □ Easy □ Relatively easy □ Normal □ Relatively difficult  □ Difficult |
| --- | --- |
| Level of satisfaction | □ Satisfied □ Relatively satisfied □ Neither satisfied nor dissatisfied  □ Relatively dissatisfied □ Dissatisfied |

- Any other comments on the tasks submitted.

7. Any other comments on the small group re-education workshops.

[Question 4] Evaluation of the re-education workshop

1. Workshop

| Frequency of workshop (once a year) | □ Few □ Relatively few □ Appropriate  □ Relatively many □ Many |
| --- | --- |
| Time taken  (about 3 hours) | □ Short □ Relatively short □ Appropriate □ Relatively long  □ Long |
| Level of understanding | □ Very understandable □ Understandable  □ Neither understandable nor not understandable  □ Not very understandable □ Not understandable |
| Usefulness | □ Useful □ Relatively useful □ Neither useful nor not useful  □ Not very useful □ Not useful |
| Level of satisfaction | □ Satisfied □ Relatively satisfied  □ Neither satisfied nor dissatisfied □ Relatively dissatisfied  □ Dissatisfied |

2. Please select a maximum of three lessons that you particularly enjoyed.

□ Lecture: Progress of the TMM CommCohort Study

□ Lecture: Progress of the TMM BirThree Cohort Study

□ Lecture: Returning genomic results and its ICP

□ Lecture: Progress of other ToMMo projects

□ Group discussion: ICP

□ Role-play: ICP

□ Questions and answers about ICP in the pre-survey

□ Modeling of the ICP by ToMMo GMRCs

□ Lecture: Posting medical records

□ Lecture: Details of a pilot study on returning genomic results

3. Please select which topics you hope to learn more about in future workshops (No limit).

□ Knowledge of epidemiology, genomes, and so on

□ Research progress by the ToMMo

□ ICP

□ ToMMo GMRC practices

□ Other ( )

4. Please select which style of learning you would prefer in future workshops (No limit).

□ Information exchange between ToMMo GMRCs

□ Lectures or training by internal researchers

□ Lectures or training by external researchers

□ Group discussions

□ Free discussion

□ Other ( )

5. Any other comments on the annual re-education workshop.

[Question 5] Quiz

Please check the relevant box.

*Please complete without assistance or referring to any text or reference book.

1. Basically, case-control studies and cohort studies are retrospective and prospective studies, respectively.

□ Yes □ No □ I don’t know

2. Case-control studies have a tendency to show selective bias and recall bias.

□ Yes □ No □ I don’t know

3. The genome is located in cells and acts like a blueprint for organisms.

□ Yes □ No □ I don’t know

4. In the double helix structure of DNA, one chain contains paternal information and the other contains maternal information.

□ Yes □ No □ I don’t know

5. The position of a specific gene on a chromosome is roughly determined.

□ Yes □ No □ I don’t know

6. It is possible for children with a genetic disease to be born from healthy parents.

□ Yes □ No □ I don’t know

7. Lifestyle-related diseases, atopic dermatitis, asthma, and so on are polygenic diseases.

□ Yes □ No □ I don’t know

8. Multiple genetic polymorphisms have been found in GWAS as determinants of common diseases and relevant personalized prevention has already been applied.

□ Yes □ No □ I don’t know

9. To obtain informed consent from potential participants, GMRCs should fairly explain both the advantages and disadvantages of the research.

□ Yes □ No □ I don’t know

10. GMRCs should respect participants’ autonomy and not force consent.

□ Yes □ No □ I don’t know

[Question 6] Any other comments on working as a ToMMo GMRC.

Date completed: (yy/mm/dd)

Thank you for your cooperation. Your answers will be used to help improve the education and training program for ToMMo GMRCs.
