# Supplementary material for: A training and education program for genome medical research coordinators in the genome cohort study of the Tohoku Medical Megabank Organization
Source: BMC Med Educ. 2019 Aug 2;19:297. doi: 10.1186/s12909-019-1725-5 (PMC6679441; doi:10.1186/s12909-019-1725-5)
Supplement: Supplementary file 4 — Table S2. Evaluation of the 17 lectures in the initial education and training program according to the participants’ demographics. (DOCX 25 kb) [file 12909_2019_1725_MOESM4_ESM.docx]

**Additional Table 2 Evaluation of the 17 lectures in the initial education and training program according to the participants’ demographics**

|  | Age  (n = 94) | | Educational background  (n = 94) | | |  | Medical qualification  (n = 93) | |
| --- | --- | --- | --- | --- | --- | --- | --- | --- |
|  | <40 | ≥40 |  | University/Graduate school | others |  | Nurse/Clinical laboratory technologist | others |
| Time taken | n (%) |  |  | n (%) |  |  | n (%) |  |
| Short/relatively short | 5 (5.3) | 11 (11.7) |  | 1(1.1) | 15 (16.0) |  | 7 (7.5) | 9 (9.7) |
| Appropriate | 18 (19.2) | 38 (40.4) |  | 15 (16.0) | 41 (43.6) |  | 23 (24.7) | 32 (34.4) |
| Relatively long/long | 6 (6.4) | 16 (17.2) |  | 6 (6.4) | 16 (17.0) |  | 11 (11.8) | 11 (11.8) |
|  |  |  |  |  |  |  |  |  |
| Level of difficulty |  |  |  |  |  |  |  |  |
| Relatively easy/neither easy nor difficult | 10 (10.6) | 24 (25.5) |  | 9 (9.6) | 25 (26.6) |  | 20 (21.5) | 13 (14.0)  * |
| Relatively difficult | 19 (20.2) | 33 (35.1) |  | 11 (11.7) | 41 (43.6) |  | 20 (21.5) | 32 (34.4) |
| Difficult | 0 (0.0) | 8 (8.5) |  | 2 (2.1) | 6 (6.4) |  | 1 (1.1) | 7 (7.5) |
|  |  |  |  |  |  |  |  |  |
| Confidence gained |  |  |  |  |  |  |  |  |
| Gained confidence | 16 (17.0) | 35 (37.2) |  | 13 (13.9) | 38 (40.4) |  | 22 (23.7) | 28 (30.1) |
| Neither gained confidence nor did not gain confidence | 12 (12.8) | 19 (20.2) |  | 6 (6.4) | 25 (26.6) |  | 15 (16.1) | 16 (17.2) |
| Little confidence or no confidence gained | 1 (1.1) | 11 (11.7) |  | 3 (3.2) | 9 (9.6) |  | 4 (4.3) | 8 (8.6) |
|  |  |  |  |  |  |  |  |  |
| Level of satisfaction |  |  |  |  |  |  |  |  |
| Satisfied/relatively satisfied | 20 (21.3) | 41 (43.6) |  | 15 (16.0) | 46 (48.9) |  | 29 (31.2) | 31 (33.3) |
| Neither satisfied nor dissatisfied | 9 (9.6) | 16 (17.0) |  | 6 (6.4) | 19 (20.2) |  | 9 (9.7) | 16 (17.2) |
| Relatively dissatisfied | 0 (0.0) | 4 (4.3) |  | 0 (0.0) | 4 (4.3) |  | 2 (2.2) | 2 (2.2) |
| No answer | 0 (0.0) | 4 (4.3) |  | 1 (1.1) | 3 (3.2) |  | 1 (1.1) | 3 (3.2) |

**p* = 0.0266 (Fisher’s exact test).
